# Genetics and epigenetics of pediatric leukemia in the era of precision medicine

**DOI:** 10.12688/f1000research.14634.1

**Published:** 2018-07-18

**Authors:** Kristie N Ramos, Irma N Ramos, Yi Zeng, Kenneth S Ramos

**Affiliations:** 1 Department of Medicine, Division of Pulmonary, Allergy, Critical Care and Sleep Medicine, University of Arizona College of Medicine-Tucson, Tucson, USA; 2 Department of Promotion Health Sciences, University of Arizona Mel and Enid Zucherman College of Public Health, Tucson, USA; 3 Department of Pediatrics, Division of Pediatric Hematology/Oncology, University of Arizona College of Medicine-Tucson, Tucson, USA; 4 University of Arizona Cancer Center, Tucson, USA; 5 Department of Medicine, Division of Clinical Support and Data Analytics, University of Arizona College of Medicine-Phoenix, Phoenix, USA

**Keywords:** Epigenetics, Precision Medicine, Pediatric Leukemia

## Abstract

Pediatric leukemia represents a heterogeneous group of diseases characterized by germline and somatic mutations that manifest within the context of disturbances in the epigenetic machinery and genetic regulation. Advances in genomic medicine have allowed finer resolution of genetic and epigenetic strategies that can be effectively used to risk-stratify patients and identify novel targets for therapy. This review discusses the genetic and epigenetic mechanisms of leukemogenesis, particularly as it relates to acute lymphocytic leukemias, the mechanisms of epigenetic control of leukemogenesis, namely DNA methylation, histone modifications, microRNAs, and LINE-1 retroelements, and highlights opportunities for precision medicine therapeutics in further guiding disease management. Future efforts to broaden the integration of advances in genomic and epigenomic science into the practice of pediatric oncology will not only identify novel therapeutic strategies to improve clinical outcomes but also improve the quality of life for this unique patient population. Recent findings in precision therapeutics of acute lymphocytic leukemias over the past three years, along with some provocative areas of epigenetics research, are reviewed here.

## Impact statement

Despite major advances in the treatment of pediatric leukemias, the etiology of pediatric leukemia remains largely unknown. Recent advances in molecular genetics and genomic medicine are discussed here that exemplify progress in the molecular classification of this group of diseases, the genetic and epigenetic strategies to risk-stratify patients, and the optimization of precision therapies to target initiator lesions and biochemical pathways involved in leukemogenesis. Although significant advances have been made, much work remains to be done to fully realize the power of precision approaches and therapeutics in the treatment of pediatric leukemias.

## Pediatric leukemias

Pediatric leukemias result from germline and somatic mutations that act in concert with disturbances of the epigenetic machinery to give rise to altered phenotypes. Despite the large variety of mutations characterized to date and evidence pointing to the involvement of immune system defects in the context of environmental exposures ^1^, the exact etiology of pediatric leukemia remains largely unknown. Advances in molecular genetics and genomic medicine now allow a more precise and comprehensive classification of this group of diseases, finer resolution of genetic and epigenetic strategies to risk-stratify patients, and optimization of precision therapies to target the initiator lesions and biochemical pathways involved in leukemogenesis. Given that acute lymphoblastic leukemia (ALL) is the most common pediatric malignancy ^2^, this review will focus on ALL unless otherwise noted.

Genomic profiling of ALL samples has identified a constellation of structural rearrangements, submicroscopic DNA copy number alterations, and sequence mutations ^3^, and a large degree of heterogeneity in molecular deficits is associated with ALL at diagnosis and relapse. For example, the acquisition of additional high-risk genetic mutations across all genetic subtypes in relapsed ALL clearly differentiates genetic profiles relative to time of original diagnosis ^4^. Nearly 20% of relapsed ALL specimens contain mutations in CREB-binding protein (CREBBP), a genetic deficit associated with changes in histone acetylation/deacetylation ^5^, and cases of relapsed ALL have also been associated with mutations in KRAS, 5′-nucleotidase, cytosolic II (NT5C2), phoshoribosyl pyrophosphate synthetase 1 (PRPS1), NRAS, fms-related tyrosine kinase 3 (FLT3) receptor, and PTPN11 ^6– 9^. This genomic heterogeneity emphasizes the importance of genetic and epigenetic interactions as key determinants of clinical presentation and progression of ALL phenotypes.

## Genetic and epigenetic mechanisms of leukemogenesis

Chromosomal rearrangements create fusion gene products, and the expression of these gene products in combination with age, white blood cell count, the presence or absence of central nervous system or testicular disease (or both) at time of diagnosis, and minimal residual disease status is the basis for prognosis prediction and disease management ^5^. Sentinel chromosomal translocations occur in nearly all cases of childhood B-cell ALL and many of these represent initiating events of prognostic significance; however, a major diagnostic challenge is that some of these genetic changes are not readily detectable by cytogenetic analysis of metaphase chromosomes and require reverse transcription–polymerase chain reaction (RT-PCR) amplification or fluorescence *in situ* hybridization (FISH) for accurate detection ^10^. In some instances, BCR-ABL mutations, IKZF1 (also known as Ikaros) deletions, fusions of tyrosine kinase or CRLF1, and mutations of JAK1 and JAK2 are being tested in clinical trial settings. The availability of next-generation sequencing-based gene panels to evaluate pediatric cancers in the future may help to advance current standards of clinical practice ( https://www.businesswire.com/news/home/20160218006343/en/Children).

ETV6-RUNX1 rearrangements are of particular interest given their involvement in 25% of standard-risk childhood B-cell ALL ^10^. Interestingly, ETV6-RUNX1 mutations can be detected in samples from patients who do not go on to develop ALL, suggesting that this translocation functions in the context of additional mutations to become pathogenic ^11^. Rearrangements of KMT2A (also known as mixed lineage leukemia 1, or MLL1) are also of interest given that over 100 fusion partners have been identified which combine sequence alterations with loss of epigenetic control through disruption of lysine methyltransferase activity ^12, 13^. KMT2A rearrangements occur in about 75% of infants with B-cell ALL, especially those less than six months of age, and in 2% of older children, adolescents, and adults with ALL ^13^. Additionally, FLT3 is commonly overexpressed in infant patients with KMT2A rearrangements ^14^, but the use of FLT3 inhibitors has not resulted in advantageous clinical outcomes ^15^. Thus, current efforts are focused on drug candidates targeting histone deacetylases (HDACs) or methyltransferases as possible therapeutic agents (ClinicalTrials.gov Identifiers: NCT02141828, NCT01483690, NCT01321346, and NCT02828358) ^10^.

The BCR-ABL1 rearrangement resulting from the t(9;22)(q34;q11.2) fusion (that is, Philadelphia chromosome) is present in nearly all chronic myelocytic leukemia cases as well as a subset of patients with ALL (3 to 5% of childhood B-cell ALL) ^10^. Ph ^+^ ALL cases oftentimes carry deletions in IKZF1 and PAX5 (also known as paired box 5) transcription factors, both of which are involved in the regulation of B-cell development ^16^. Studies to evaluate the efficacy of imatinib—an ABL tyrosine kinase inhibitor (TKI)—in combination with intensive chemotherapy in children with Ph ^+^ ALL have reported dramatic improvements in overall survival ^17– 19^. However, because long-term imatinib therapy can result in ABL tyrosine kinase domain point mutations that afford decreased TKI sensitivity, additional research into effective combination therapies that can specifically target molecular deficits is needed ^20, 21^.

A BCR-ABL1–like ALL has also been described with activated kinase expression profiles that closely resemble Ph ^+^ ALL but that also involve unregulated activation of cytokine signaling pathways and mutation of B cell–associated transcription factors ^16, 22, 23^. Translocation of “Abelson kinase (ABL) class” genes such as ABL1, ABL2, colony-stimulating factor 1 receptor (CSF1R), and platelet-derived growth factor receptor beta (PDGFRB) results in fusion proteins that activate receptor tyrosine kinase or non-receptor tyrosine kinase signaling ^23^. A very high frequency of *IKZF1* deletions, fusions of tyrosine kinase genes, fusions of *CRLF2*, and mutations of *JAK1* and *JAK2* have been detected in patients with Ph-like ALL ^16, 23^. High-risk B-ALL patients who are Ph-like have a predicted five-year event-free survival of less than 60% ^24^. Since tyrosine kinase fusion partners, including *ABL1*, *ABL2*, *CSF1R*, *PDGFRB*, and *FGFR*, respond well to the addition of TKIs ^23^, introducing TKI therapy after induction chemotherapy may improve survival in patients with Ph-like ALL. The safety and efficacy of dasatinib in combination with standard conventional chemotherapy in treating Ph-like ALL in children are currently being tested in a Children’s Oncology Group clinical trial (ClinicalTrials.gov Identifier: NCT02883049).

Although genetic and epigenetic profiling data on T-cell ALL are not as abundant as what has been published on B-cell ALL, Furness *et al*. recently demonstrated interesting findings in regard to the evolution of genetic alterations in STIL-TAL1 + T-cell ALL ^25^. Using single-cell multicolor FISH, Furness *et al*. found that both STIL-TAL1 fusion and loss of both CDKN2A alleles were present in the earliest detectable leukemic subclones but that other alterations such as NOTCH1 and PTEN mutations appeared to be secondary events. These findings are of significance given that (1) these mutational events can serve as potential markers for minimal residual disease monitoring and (2) the TAL1 regulatory complex could be a future target for therapy ^25^. Further studies are needed to examine the genetic and epigenetic complexity that exists among T-cell ALL subtypes.

## Molecular mechanisms of epigenetic control in leukemogenesis

Many of the chromosomal rearrangements that alter cellular differentiation and result in leukemogenesis do so by interfering with epigenetic mechanisms or the epigenetic machinery or both ^26, 27^. As such, a brief discussion of the molecular deficits in epigenetic control within the context of leukemogenesis is warranted. Epigenetic mechanisms encompass a repertoire of heritable alterations in gene expression that occur in the absence of changes to the DNA sequence. In the case of leukemias, epigenetics carries added significance given that B- and T-cell maturation is associated with changes in DNA structure that allow these cells to differentially recognize molecules and that, by necessity, will require further fine-tuning through the epigenetic machinery. Transient changes in the epigenetic machinery, as seen upon binding of transcription factors to DNA or the reversible acetylation of histones to mediate chromatin unwinding, are not heritable. Instead, epigenetics is concerned mainly with covalent DNA and histone modifications that are replicated in daughter cells upon cell division. The placement, removal, and interpretation of these marks are denoted in the literature as writers, erasers, and readers, respectively, to identify the complex set of enzymes that catalyze these reactions.

## DNA methylation

DNA methylation is a pivotal component of cellular differentiation, gene expression, and genome-wide maintenance and stability and is currently recognized as the most prevalent epigenetic modification in the development of ALL. Methylation within the 5′–3′ cytosine guanine (CpG) dinucleotide sequence has been most studied, particularly in regions of the genome having a GC content greater than 50% and thus referred to as CpG islands ^28, 29^. These islands are generally found in the 5′ region of genes and are specifically involved in regulation of gene expression. DNA methyltransferases (DNMTs) (1, 2, 3a, and 3b) are the enzymes responsible for the transfer of methyl groups and are known to play important roles in the development and progression of cell division.

Both targeted and genome-wide alterations in DNA methylation can play key etiological roles in pediatric leukemias ^28, 30^. Initial studies have suggested that aberrant promotor methylation is associated with prognosis ^31^, cytogenetic alterations ^32^ and subtypes ^33^, and likelihood of relapse ^34^. Subsequent studies have identified hypermethylated CpG islands across various genetic and immunophenotypic leukemia subtypes, suggesting that deficits in DNA methylation are key to malignant transformation across multiple ALL subtypes ^34– 38^. Others have presented data suggesting that bidirectional allele-specific gene expression may be due to random distribution of CpG methylation ^39^. A caveat of these studies is the small number of patients examined, thus limiting the clinical generalizability of these findings.

Analysis of 137 B-cell lineage and 30 T-cell lineage childhood ALL samples using microarrays and genome-wide cytosine methylation profiling has shown that different ALL subtypes exhibit unique DNA methylation signatures that correlate with gene expression patterns ^39^. Importantly, a common epigenetic methylation signature involving signaling molecules (TIE1, MOS, CAMLG, and GPRC5C), cell cycle regulation and proliferation (MCTS1 and DGKG), RNA metabolism (PABPN1 and PABPC5), transcription factors and transcription regulators (PROP1, TAF3, H2AFY2, ELF5, ZBTB16, CNOT1, and TADA2A), and homeobox genes (HOXA5 and HOXA6) was identified in all cases of ALL examined ^39^. This analysis compared all leukemia samples against a cohort of normal B cells at various stages of differentiation, confirming that the methylation signature could not be attributed to differentiation status alone. The common epigenetic signature correlated with gene expression in 65% of the genes identified, supporting the conclusion that alterations in cytosine methylation likely impact leukemogenesis.

Although survival is relatively high for patients with ALL managed in accordance with established protocols, the prognosis for relapsed patients remains a major clinical challenge ^40^. Thus, efforts to identify biomarkers of relapse at the time of diagnosis can be of great clinical benefit and help guide future treatment modalities. In this regard, numerous studies have attempted to use DNA methylation signatures at diagnosis to predict relapse ^36, 41– 43^ but these studies have yielded variable results in small patient cohorts. In a study of 33 B-cell precursor ALL cases by Hogan *et al*., higher DNA methylation levels in CpG islands were measured at relapse ^44^. In another study, by Nordlund *et al*., 27 paired samples with variable ALL subtypes showed that relapse-associated CpG sites overlapped with genes regulated by transcription factors such as REST, SOX2, NANOG, and OCT4 ^36^. Conversely, a study by Kunz *et al*. examined 13 T-cell ALL paired samples and reported increased hypomethylation of promotor regions in relapsed samples ^45^. Such heterogeneity of findings likely reflects epigenetic and genomic alterations throughout disease progression and across different leukemia subtypes.

To date, alterations in DNA methylation have generally been studied in solid malignancies, and increased DNA methylation and consequent silencing have been notable in promotor regions coupled with global hypomethylation of repetitive elements and subsequent genomic instability. In sharp contrast, Bujko *et al*. ^46^ reported that repetitive elements tend to be hypermethylated in hematologic malignancies. These investigators examined overall DNA methylation status of LINE-1 and ALU elements in patients with adult acute myeloid leukemia (AML) and childhood B-cell lymphoblastic leukemia. Higher DNA methylation of LINE-1 was observed in adult AML and pediatric ALL samples compared with normal controls, and additional increased methylation of ALUs was observed in patients with B-cell ALL. Furthermore, higher methylation levels were seen in B-cell ALL samples compared with both AML and control samples, and a positive correlation was seen between DNA methylation and total leukocyte count ^46^. Although the significance of these findings remains to be established, the data suggest that regulatory control of repetitive sequences involved in chromosomal rearrangements is differentially regulated compared with other cancer types.

In the aforementioned study by Hogan *et al*., DNA methylation was examined in 33 matched B-cell precursor ALL samples at the time of diagnosis compared to relapse. A total of 1147 CpG sites in 905 genes showed increased methylation levels at relapse. More specifically, gene expression profiles also differed for early relapse (less than 36 months) versus late relapse (greater than or equal to 36 months). Many of the unique targets were genes within the Wnt signaling cascade ^44^ and were subsequently found to be responsive to decitabine ^47^, potentially implicating the Wnt pathway in relapsed disease. These data speak to the role of epigenetic modifications in leukemic disease progression and again suggest the possible use of DNA methylation inhibitors as therapeutic agents.

## Covalent histone modifications

Histone tail modifications (such as acetylation, methylation, phosphorylation, sumoylation, and ubiquitylation) can alter gene expression, and specific marks and positions within the N-terminal tail are linked to either transcriptional activation or repression ^48^. Histone modifications are catalyzed by various enzymes, including histone lysine demethylases (HKDMs), histone acetyltransferases (HATs), and HDACs, which in coordination with other DNA-binding proteins help to define chromatin architecture. Four core histone proteins (H2A, H2B, H3, and H4) form a basic scaffolding structure called a nucleosome, and post-translational modifications of histone tails go on to define chromatin architecture and control chromatin accessibility. Acetylation and methylation are stable modifications that regulate gene expression, and modifications such as these are subject to epigenetic control. Other modifications such as phosphorylation, sumoylation, and ubiquitylation represent transient modifications of functional significance. The combination of both stable and transient histone modifications is often referred to as the “histone code” that defines the terms of transcriptional regulation for the genome.

To date, a number of studies have implicated covalent histone modification in the development of pediatric ALL. For example, a t(4;11) translocation results in the creation of the KMT2A-AF4 fusion gene, which is the most common KMT2A rearrangement identified to date in infant ALL ^40^. This fusion protein binds fewer genomic regions than KMT2A wild-type, leading to abnormal DNA methylation and extensive chromatin remodeling ^49^. However, not all infant ALL samples contain KMT2A rearrangements and thus studies have sought to examine the genetic abnormalities in these cases. As such, a BRD9-NUTM1 fusion gene resulting from t(5;15)(p15;q14) was described in two KMT2A-r–negative infant ALL cases ^12, 50^. Additionally, BRD9 is a bromodomain-containing protein that likely functions in chromatin remodeling and that has recently been implicated in AML ^51^, while NUTM1 enhances the HAT activity of EP300, a CREBBP homolog known to fuse with BRD4 in NUT midline carcinoma ^52^. Furthermore, recent transcriptome sequencing studies have found other NUTM1 fusion genes in severe sporadic ALL cases (including IKZF1-NUTM1, AFF1-NUTM1, and ZNF618-NUTM1) ^53, 54^. The development of innovative personalized therapies based on the use of DOT1L inhibitors for the treatment of pediatric and adult MLLr has shown encouraging results ^55^. DOT1L catalyzes the mono-, di-, and tri-methylation of H3K79 within the ordered core of H3 and is required to initiate tumorigenesis and maintain the malignant phenotype of MLLr ^56^. Thus, DOT1L inhibitors hold considerable promise in the clinical management of leukemia.

In other studies, loss-of-function mutations in the CREBBP gene located on chromosome 16 have been observed in 18% of relapsed ALL cases as well as early T-cell precursor ALL ^57^. Zinc finger protein 284 (ZNF384) rearrangements have recently been described in a new subtype of B-cell precursor ALL, in which up to 80% of patients have a mutation of translocation involving an epigenetic regulating gene ^54, 58^. Importantly, fusion of ZNF384 with either CREBBP or EP300 results in dominant-negative loss of histone lysine acetyltransferase activity and global reduction of histone acetylation and subsequently increases sensitivity to HDAC inhibitors *in vitro* ^53^. This emerging pattern implicates coordinated regulation between chromosomal rearrangements and epigenetic dysregulation as pivotal events in leukemogenesis. This interpretation opens the door for development of significant advances in precision diagnosis and treatment for pediatric leukemia involving ETV6-CBX3, RUNX1-ASXL1, and NOL4L-ASXL1 fusions ^49, 53^.

## MicroRNA mechanisms

Altered microRNA (miRNA) expression has the ability to effect several known regulatory pathways in ALL pathogenesis ^59– 62^. miRNAs are small non-coding RNA molecules that function in transcriptional and post-transcriptional regulation of gene expression via base pairing with complementary sequences. The interaction of miRNAs with their target mRNAs results in cleavage of the mRNA strand, destabilization through poly(A) shortening, or deficits in translation or a combination of these. Although most of what we now understand about miRNA biology defines intracellular events, evidence establishing an important role for miRNAs as extracellular, circulating signaling molecules is fast accumulating ^63^. Over 1000 known miRNAs have been identified in the human genome ^64^, and of the miRNAs implicated in ALL, miR-34, miR-128, miR-142, and miR-181 are overexpressed ^59, 65, 66^ whereas miR-100 and miR-196b are underexpressed ^61, 65^.

A study by Schotte *et al*. comparing 397 miRNAs in 81 pediatric ALL cases against 17 normal CD34 ^+^ stem cell controls reported unique miRNA signatures for various ALL subtypes and found miR-143 and miR-140 to be 70- to 140-fold lower in B-cell ALL samples versus control ^67^. These investigators also examined the impact of miRNA expression on chemotherapy responsiveness and found that decreased miR-454 expression was associated with L-asparaginase resistance but that increased expression of miR-99, miR-100, and miR-125b was associated with vincristine and daunorubicin resistance. Furthermore, eight miRNAs (miR-10a, -134, -214, -484, -572, -580, -624, and -627) were associated with longer event-free survival, leading the team to hypothesize that those associated with increased event-free survival likely had tumor suppressor functions exerted via apoptotic signaling (miR-10a), inhibition of proliferation (miR-10a and -214), and downregulation of SOX2 (miR-134) ^67^. These findings have opened the door for the design of precision diagnostics where patients are screened for the presence of these miRNAs at time of diagnosis to define an optimal chemotherapy regimen for their disease. Furthermore, in cases where miRNA expression is repressed by hypermethylation ^65, 68– 71^, a DNMT inhibitor regimen could be implemented to reverse epigenetic modifications and improve survival.

## Transposable elements and leukemias: unanswered questions

Little is known about the role of transposable elements in leukemogenesis, and most efforts to date have focused strictly on AML. A previous report on ALL has implicated translocation junctions at the transcription factor 3 (TCF3)/E2A immunoglobulin enhancer-binding factors E12/E47 (E2A) locus clustered within, or in proximity to, transposable element sequences ^68^. Transposable elements make up 45% of the human genome ^69^. Given the important role of these elements in regulating the transcriptional activity of genes, there is interest in determining their putative role in leukemogenesis. LINEs, abundant retrotransposons in the human genome, are major sites of epigenetic control because of the high density of CpG islands contained within these sequences. These elements influence gene expression via several mechanisms; the most notable and probably best understood is their ability to induce insertion mutations into open reading frames or intronic regions following a full cycle of retrotransposition.

Early studies of the life cycle of L1 retroelements established a causal relationship between L1 insertion mutations and cancer ^70^. More recently, data on hypomethylation-mediated reactivation of LINEs have prompted investigations aimed at elucidating the role of LINEs in the regulation of chromatin dynamics and genome stability across the full spectrum of human development. At its most fundamental level, L1 mobilization can disrupt local genome architecture, induce DNA strand breaks, mediate alternative splicing, increase the frequency of recombination, and induce loss of transcriptional control of neighboring genes. Most L1 insertions are truncated at the 5′ end and carry insertions/deletions that render these newly inserted elements unable to retrotranspose ^71^.

Previous work has established that genetic ablation of RB proteins leads to reactivation of L1 retroelements ^16, 70^. pRB interacts with HDAC1, DNMT1, pRB-associated protein 48 (RbAp48), suppressor of variegation 3-9 homolog 1 ( *Drosophila*) (Suv39H1), and suppressor of variegation 4-20 homolog 2 ( *Drosophila*) (Suv420H2) to induce signatures of epigenetic silencing ^16^. Epigenetic reactivation of L1 by DNA-damaging agents involves proteasomal-mediated degradation of DNMT1 and loss of RB-mediated silencing ^72, 73^. These findings are of potential relevance given previous reports implicating Rb-1 as a prognostic factor in pediatric ALL ^74^.

Rb-1 is normally expressed in hematopoietic cells and inactivated by point mutations with predominance for exons 20–24 in various cancers. In these studies, bone marrow from 26 pediatric patients with leukemia (18 ALL and eight AML) was studied. In ALL cases, two samples in exon 20 (11.11%), one in exon 21 (5.56%), and four in exon 22 (22.22%) had altered conformation. All but one of these cases were classified as high-risk leukemia patients who either relapsed or never achieved remission. In addition to having the ability to disrupt the architecture of the genome, LINEs regulate gene expression patterns in cells via epigenetic mechanisms. Thus, leukemia may involve derangements in LINE-1 expression that compromise genome integrity and function. This hypothesis has never been rigorously examined.

## Perspectives for the precision medicine era

Molecular signatures defined on the basis of DNA methylation, covalent histone modifications, and miRNA expression profiles can help to better stratify disease phenotypes both at the time of diagnosis and during the course of disease progression. Furthermore, these molecular signatures can optimize medical therapies designed to target specific molecular deficits and minimize adverse reactions. These are the foundational underpinnings of precision medicine, and although significant advances have been realized in the clinical management of several solid and liquid tumor malignancies, more remains to be done in the diagnosis and treatment of pediatric leukemias. Future avenues of research include systematic mapping of epigenetic modifications on a genome-wide scale in primary ALL cells to assess the impact of somatic mutations on cellular programming and, consequently, cellular behavior. In this context, the Blueprint Consortium (which maps human blood cell epigenomes) has contributed genome-wide data on histone modification in primary ALL cells from 15 patients with B-cell precursor ALL ^75^. Use of these data can help establish a regulatory mechanism for hereditary risk of high hyperdiploidy ALL, a hypothesis put forth by the consortium to evaluate a risk allele within the enhancer element on chromosome 10p21.2 that disrupts RUNX3 binding, decreases ARID5B expression, and arrests normal lymphocyte development to initiate leukemogenesis ^76^.

Deregulated H3K9ac ^77^, global acetylation ^78^, and increased HDAC activity ^79^ occur in ALL cells, and epigenetic modifications are more commonly observed in relapsed ALL cases verses those at the time of diagnosis. Although we do know that epigenetic alterations are likely implicated in the initiation, development, and relapse of pediatric ALL, most epigenetic trials to date have been completed in adults and thus major knowledge gaps persist in regard to how this information translates to pediatric ALL cases. To date, the analysis of DNA methylation has proven valuable in determining leukemic cell origin and cytogenetic features and can be developed as a biomarker of ALL in the future. The challenge remains to understand how these and other epigenetic alterations operate within the framework of gene fusions and other somatic mutations in ways that can be targeted for precision medicine intervention.

The constitutive activation of kinase signaling pathways in ALL suggests that the development of kinase inhibitors that target specific molecular deficits may be of value in the precision medicine space. PDGFRB rearrangements commonly occur in patients who fail to respond to induction chemotherapy (greater than or equal to 25% residual disease). As such, this mutation can be screened for in cases where conventional therapies are of diminished value. To this end, current clinical trials are examining the use of dasatinib or ruxolitinib in combination with chemotherapy for patients with ABL-class fusions or JAK signaling pathway alterations, respectively ^80^.

Whole genome testing could be considered during induction therapy in order to identify appropriate clinical trials for these patients. The utility of this approach can be exemplified in patients with trisomy 21–associated ALL. These patients carry a 10-fold higher risk of developing ALL (almost always of B-cell lineage) in addition to an increased risk of chemotherapy toxicity ^81^. As many as 50 to 60% of Down syndrome–associated ALL cases have CRLF2 rearrangements, most commonly characterized as a P2RY8-CRLF2 fusion due to deletion of the pseudoautosomal region of the sex chromosomes ^82^. Fifty percent of CRLF2-rearranged Down syndrome–associated ALL cases have concomitant JAK mutations (commonly a JAK2 R683G point mutation) ^83^, but JAK inhibitors have yet to be formally evaluated in clinical trials.

Activating mutations in RAS pathway genes have also been identified in several pediatric ALL subtypes, including high hyperdiploid and hypodiploid ALL, infant ALL, and certain cases of Ph-like ALL ^84, 85^. Genes implicated include GTPases, KRAS, NRASM, HRAS, protein tyrosine phosphatase non-receptor type 11 (PTPN11), casitas B-lineage lymphoma (CBL), and FLT3. Further research is needed to elucidate the causal relationships in the context of leukemogenesis that would be of significance given that these gene targets would be suitable for the application of precision therapy.

The importance of precision approaches in the clinical management of pediatric leukemias is emphasized by the high frequency of chemoresistance-associated mutations during the course of therapy and the potential role of predisposing genetic polymorphisms in determining disease onset, progression, and response to treatment. Continued translational and clinical research is needed to further define (1) the evolving genomic complexity of the disease and (2) the need for stratification of a highly heterogeneous group of diseases. At the core of precision approaches in cancer is the need for better resolution of molecular phenotypes and the identification of mechanism-based and mechanism-agnostic approaches to prognosis, diagnosis, and clinical management of the disease.

A significant limitation at present is the need for coordinated drug discovery and drug repurposing programs that can help identify small molecules and biological approaches to therapy. Another challenge is the fact that most studies to date focus on the use of single agents in poor-phenotype cohorts. As such, concerted efforts are needed to evaluate combination therapies in a systematic fashion and to better match treatment to molecular phenotypes across a highly heterogeneous group of patients, and longitudinal follow-up is necessary to evaluate disease-free survival and long-term risks associated with therapy. Although significant advances have been made in this space, much work remains to be done in pediatric oncology to fully realize the power of precision approaches and therapeutics.
